# An Intra-Vaginal Zinc Oxide Tetrapod Nanoparticles (ZOTEN) and Genital Herpesvirus Cocktail Can Provide a Novel Platform for Live Virus Vaccine

**DOI:** 10.3389/fimmu.2019.00500

**Published:** 2019-03-20

**Authors:** Alex Agelidis, Lulia Koujah, Rahul Suryawanshi, Tejabhiram Yadavalli, Yogendra Kumar Mishra, Rainer Adelung, Deepak Shukla

**Affiliations:** ^1^Department of Ophthalmology and Visual Sciences, University of Illinois, Chicago, IL, United States; ^2^Department of Microbiology and Immunology, University of Illinois, Chicago, IL, United States; ^3^Institute for Materials Science, Kiel University, Kiel, Germany

**Keywords:** herpes simplex virus, genital herpes, immunotherapy, live virus vaccine, viral infection

## Abstract

Herpes simplex virus type-2 (HSV-2) is a common cause of genital infections throughout the world. Currently no prophylactic vaccine or therapeutic cure exists against the virus that establishes a latent infection for the life of the host. Intravaginal microbivac is a developing out-of-the-box strategy that combines instant microbicidal effects with future vaccine-like benefits. We have recently shown that our uniquely designed zinc oxide tetrapod nanoparticles (ZOTEN) show strong microbivac efficacy against HSV-2 infection in a murine model of genital infection. In our attempts to further understand the antiviral and immune bolstering effects of ZOTEN microbivac and to develop ZOTEN as a platform for future live virus vaccines, we tested a ZOTEN/HSV-2 cocktail and found that prior incubation of HSV-2 with ZOTEN inhibits the ability of the virus to infect vaginal tissue in female Balb/c mice and blocks virus shedding as judged by plaque assays. Quite interestingly, the ZOTEN-neutralized virions elicit a local immune response that is highly comparable with the HSV-2 infection alone with reduced inflammation and clinical manifestations of disease. Information provided by our study will pave the way for the further development of ZOTEN as a microbivac and a future platform for live virus vaccines.

## Introduction

Herpes simplex virus-2 (HSV-2) is a neurotropic double stranded DNA virus capable of lytic infection in multiple host cell types as well as latent infection in neuronal cells (1). The viral DNA genome is encased in an icosadeltahedral protein capsid which is surrounded by tegument proteins (2). The capsid and tegument are enveloped in a lipid bilayer composed of multiple viral proteins and glycoproteins on the surface of the virus particle (3). HSV-2 entry into the host cell primarily involves the interaction of the viral entry glycoproteins with various cell surface receptors that facilitate virion envelope fusion with the plasma membrane of the host cell causing capsid penetration into the cytoplasm (4). Once the genome reaches the nucleus, viral protein production occurs in a sequential manner beginning with immediate early gene products that promote immune evasion and neurovirulence (5). Early proteins are then synthesized which are required for viral DNA replication. This is followed by production of late proteins, providing structural components of the capsid that are necessary for viral egress. HSV spreads rapidly to neighboring cells as well as the dorsal root ganglia where it establishes latency (6).

Primary infection of HSV-2 results in a variety of prolonged clinical manifestations, ranging from genital ulcerations to more severe cases like meningitis (7). While HSV-2 infection most commonly occurs in the genitalia, it may also result in oral, ocular and neurologic infections (8). In addition, genital ulcerations caused by HSV-2 and viral shedding have been definitively linked to an increased risk for acquisition of human immunodeficiency virus (HIV) infection (9, 10). HSV-2 infects over 400 million people worldwide and is one of the most common sexually transmitted infections (11). Despite its high prevalence, no cure or vaccination has been developed. Acyclovir, a nucleoside analog, is widely used to treat primary HSV-2 infection and has shown to be an efficacious therapeutic in most cases. However, acyclovir resistant strains have also evolved and treatment options are limited in those cases (12–14).

A traditional antiviral may not be the best choice since diverse response from infected patients has been observed with variations in episodes of viral shedding due to the varying degrees of localized immune response among individuals. Roughly 80% of HSV-2 seroprevalent persons are asymptomatic and report no genital lesions even with detection of viral genomes at the site of infection (15). Upon infection in immunocompetent individuals, the virus is rapidly contained by a prompt innate immune response and further suppressed by resident memory HSV-specific T cells (16–18). For the large majority of HSV-2 infected individuals, cell-mediated immune responses are able to control and protect against clinical recurrences and genital lesion development (19). While the majority of currently prescribed antivirals target the virus itself, the development of an antiviral or immunotherapeutic that inhibits infection and at the same time, facilitates a protective immune response can better guard the host against the deleterious effects of primary HSV-2 infection as well as recurrences (20–22). Alternatively, since subunit vaccines have failed to show real promise in clinical trials, a safe live virus vaccine may provide a better solution (23).

Previously, our group discovered a novel microbicidal and vaccine-like (or microbivac) platform against primary and secondary female genital herpes infections (24). The dual microbivac platform was demonstrated through the ability of uniquely designed zinc oxide tetrapod nanoparticles (ZOTEN) with engineered oxygen vacancies to strongly trap HSV-2 virion, neutralize the virus and prevent cell entry in the vaginal epithelium (25, 26). ZOTEN showed to be an effective suppressor of HSV-2 genital infection in female BALB/c mice with apparent reduction of clinical signs of vaginal infection and decreased animal mortality. ZOTEN therapy ultimately was found to create a platform for viral antigen presentation and therefore was presented as a novel microbivac with the potential to prevent primary infection and viral shedding (27). Interestingly, treatment of ZOTEN was found to have adjuvant-like properties, enhancing immunity against the virus in mice (24). The proposed mechanism for this is that ZOTEN acts to capture the virus, allowing for detection by immune cells which in turn results in enhanced T cell-mediated and antibody-mediated responses to infection and thereby suppressing a reinfection. ZOTEN's ability to target the virus particle and manipulate the host immune system demonstrates its novel and multifunctional antiviral properties with promising prophylactic and therapeutic effects (28).

In this article, we aim to better understand the vaginal immune responses and antiviral benefits of a short-term acute infection in female BALB/c mice using a ZOTEN/HSV-2 cocktail. Such a cocktail could provide more information on the microbivac benefits of ZOTEN while demonstrating its promise as a unique platform for live virus vaccine development. Our tissue specific analyses show that the cocktail inhibits infection but generates a local immune response that is highly comparable to the infection with the virus alone. It also shows the promise that ZOTEN can be given alongside to reduce the possibility of infection via any live virus vaccine.

## Materials and Methods

### Mouse Model of Genital Herpes Infection

Animal care and procedures were performed in accordance with institutional and NIH guidelines and approved by the Animal Care Committee at the University of Illinois at Chicago. Six to Eight-weeks-old female BALB/c mice obtained from Charles River Laboratories were injected with 0.1 mL medroxyprogesterone acetate (Depo-Provera) (Greenstone) to synchronize estrous cycles. Seven days after injection, mice were inoculated with HSV-2, or mock infected, with or without ZOTEN. HSV-2 strain 333 was used for all experiments. Synthesis and use of ZOTEN in antiviral assays have been described previously (24, 26, 29). ZOTEN cocktail treatment consisted of preincubating HSV-2 (or mock) in PBS for 30 min at room temperature with or without 0.1 mg/mL ZOTEN and then inoculating female mice genitals with respective solution. Each infected mouse received a viral inoculum of 5 × 10^5^ pfu in a 10 μL volume. Untreated mice received virus that was similarly incubated at room temperature.

### Synthesis of ZOTEN (Tetrapod-Form ZnO Micro-Nanoparticles)

Nanoparticles were synthesized and characterized according to our previously published studies (24). Spherical zinc microparticles, polyvinyl butyral (PVB) powder, and ethanol were obtained commercially. A mixture using these materials is prepared and burned together in the furnace at 900°C. Zn microparticles (in the form of Zn atoms, Zn dimers, Zn trimers, etc.) are generated in the flame that results from the burning of polymer PVB. In the presence of oxygen from the surrounding environment, the unstable atomic variants of Zn microparticles participate in nucleation and growth processes. Initially, Zn and O combine to form a primary cluster and once the stable nucleus has been formed, further available Zn and O atoms contribute to conventional 1D spike growth which results in growth of tetrapod-type structures. The process continues as PVB decomposes completely into CO_2_ and O_2_, resulting in an actual yield of 99.9% of ZOTEN. The formation of uniform ZnO tetrapods (ZOTEN) has been confirmed by electron microscopy; as well as the size and shape by scanning electron microscopy (30). Identical ZOTENs were used for all experiments demonstrated in this article.

### Mouse Vaginal Swabs and Detection of Virus Shedding

At days 2 and 4 post infection, mouse vaginal canal was sampled using calcium alginate swabs (Puritan, 25–800) previously dipped in OptiMEM for approximately 2 min. Swabs were performed by gently streaking vaginal canal in a circular motion 5 times and then dipping the swab into 500 μL OptiMEM. This process was performed twice for each mouse. Collected washes were briefly vortexed and centrifuged then plated on confluent monolayers of Vero cells in a plaque assay.

### Plaque Assay

Monolayers of Vero cells grown in DMEM + 10% FBS + 1% penicillin/streptomycin were washed once with PBS, then overlaid with vaginal swab washes freshly collected from mice. After incubation for 2 h, inocula were aspirated, and Vero cells were overlaid with DMEM containing 5% methylcellulose. 72 h later, cells were fixed with methanol for 10 min, media was removed, and cells were then incubated with crystal violet staining solution for 30 min to visualize plaques.

### Flow Cytometry

Mouse vaginal tissue was dissected and dissociated by incubating in 100 μL of 2 mg/mL collagenase in PBS for 4 h at 37°C. The resulting mixture was triturated with a pipet tip, suspended in an additional 1 mL of FACS buffer (5% FBS in PBS) and passed through a 70 μm filter. Cells were aliquoted into 96-well round bottom plates for staining. F_c_ receptors were blocked using TruStain FcX (101319, Biolegend) according to the manufacturer's protocol, and cells were then stained with the following antibodies from BioLegend: APC anti-mouse Gr-1 (108411), FITC anti-mouse CD45 (103107) APC anti-mouse CD3e (100311), FITC anti-mouse CD49b (103503) APC anti-mouse CD11c (117309) and PE anti-mouse F4/80 (123109). Cells were incubated with fluor conjugated primary antibodies for 1 h on ice, washed twice with FACS buffer, and analyzed with a BD Accuri C6 Plus flow cytometer. 10,000 singlet non-debris events were collected for each sample, and FlowJo X was used to process and analyze the data.

### Quantitative Polymerase Chain Reaction

Mouse vaginal tissue was dissected and dissociated by incubating in 100 μL of 2 mg/mL collagenase in PBS for 4 h at 37°C. The resulting mixture was triturated with a pipet tip, suspended in 1 mL of Trizol, and frozen at −80°C until processing. RNA extraction was performed according to Trizol manufacturer's guidelines. 2 μg of total RNA was reverse transcribed to cDNA using High Capacity cDNA Reverse Transcription kit (Thermo Fisher). Real time qPCR was performed with Fast SYBR Green Master Mix (Thermo Fisher) with the QuantStudio 7 Flex system (Life Technologies). The following mouse specific primers were used in this study:

β-actin fwd 5′-GACGGCCAGGTCATCACTATTG-3′

β-actin rev 5′-AGG AAGGCTGGAAAAGAGCC-3′

IFN-α fwd 5′-CCTGCTGGCTGTGAAAT-3′

IFN-α rev 5′-GACAGGGCTCTCCAGACTTC-3′

IFN-β fwd 5′-TGTCCTCAACTGCTCTCCAC-3′

IFN-β rev 5′-CATCCAGGCGCTGTTGT-3′

IL-1β fwd 5′-GTGGCTGTGGAGAAGCTGTG-3′

IL-1β rev 5′-GAAGGTCCACGGGAAAGACAC-3′

IL-6 fwd 5′-ACGGCCTTCCCTACTTCACA-3′

IL-6 rev 5′-CATTTCCACGATTTCCGAGA-3′

TNF-α fwd 5′-GCCTCTTCTCATTCCTGCTTG-3′

TNF-α rev 5′-CTGATGAGAGGGAGGCCATT-3′

### Mouse Tissue Histology and Staining

Mouse vaginal tissue was dissected and embedded in Tissue-Plus O.C.T. (Fisher HealthCare) then frozen on dry ice and kept at −80°C until processing. 10 μm sections were cut with a Cryostar NX50 microtome (Thermo Scientific). Sections were air dried at room temperature, fixed in ice-cold acetone for 5 min, and washed under running water for 2 min. Slides were then incubated in Mayer's Hemalum solution (EMD Millipore, 109249) for 1 min and then washed under running water for 1 min. Slides were dipped in 70% ethanol for 2 min, then in 100% ethanol for 1 min, and incubated with eosin Y alcoholic, with phloxine (Sigma, HT110316) for 1 min. Slides were then dipped in 70% ethanol for 1 min, then in 100% ethanol for 1 min, then xylene for 1 min, and coverslipped with Permount mounting medium (Thermo Fisher). Sections were visualized and photographed using a Zeiss Axioskop 2 plus microscope.

Draining inguinal lymph nodes were excised from mice at time of euthanasia and placed in 24-well plates. Lymph nodes were then photographed using a desktop scanner at 1,200 dots per inch. Lymph node areas in pixels were quantified using Adobe Photoshop CC 2018.

### Statistical Analysis

Errors bars denote SEM (*n* = 5 mice per group) unless specified otherwise. Asterisks denote significant difference by two-tailed unpaired Student's *t*-test, ^*^p < 0.05, ns or unlabeled, not significant.

## Results

### Antiviral Effects of ZOTEN/HSV-2 Cocktail at the Primary Site of HSV-2 Infection

**Figure 1:**
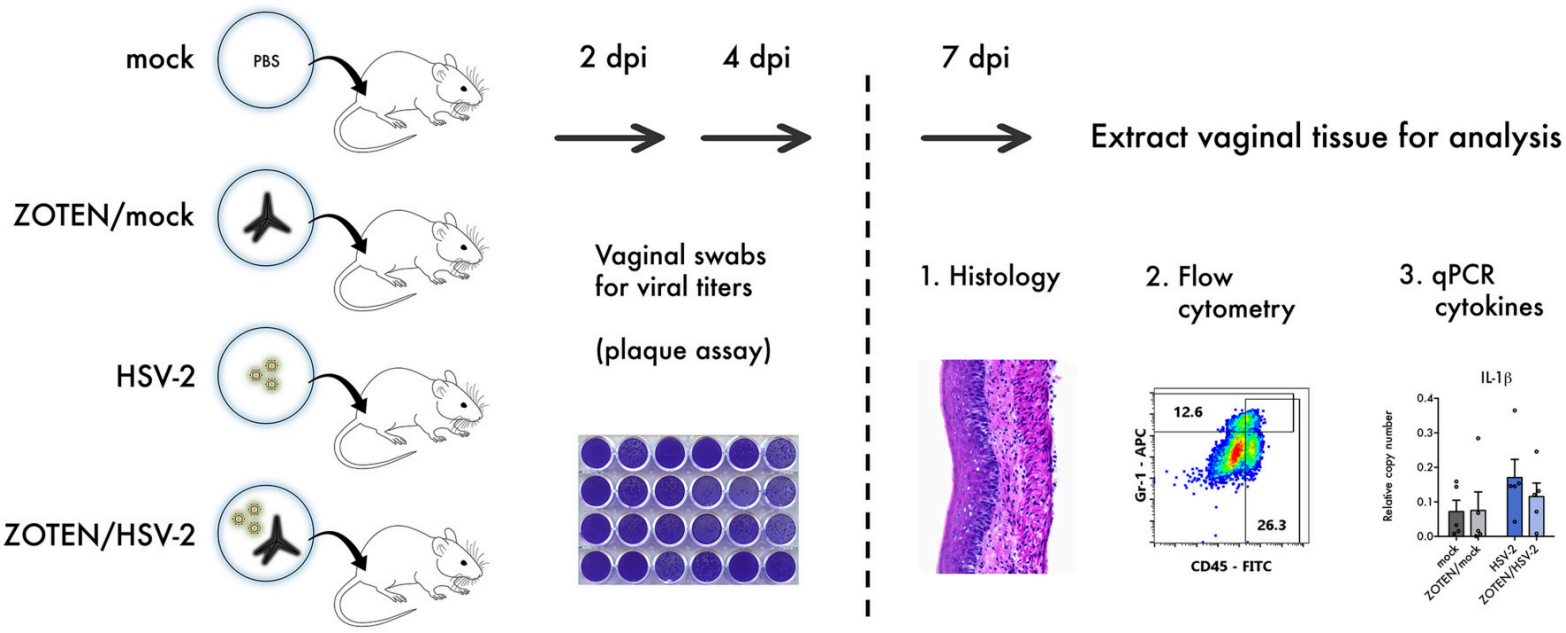
Study design. 6–8 weeks old female BALB/c mice were infected with HSV-2 or mock infected in the presence or absence of ZOTEN. At 2 and 4 days post infection (dpi), mice genitals were swabbed to detect viral shedding using a plaque assay. At 7 dpi, mice were euthanized, and vaginal tissues were extracted and analyzed by histology, flow cytometry, and quantitative PCR (qPCR) to appreciate differences in cellular infiltration and local inflammation.

**Figure 2:**
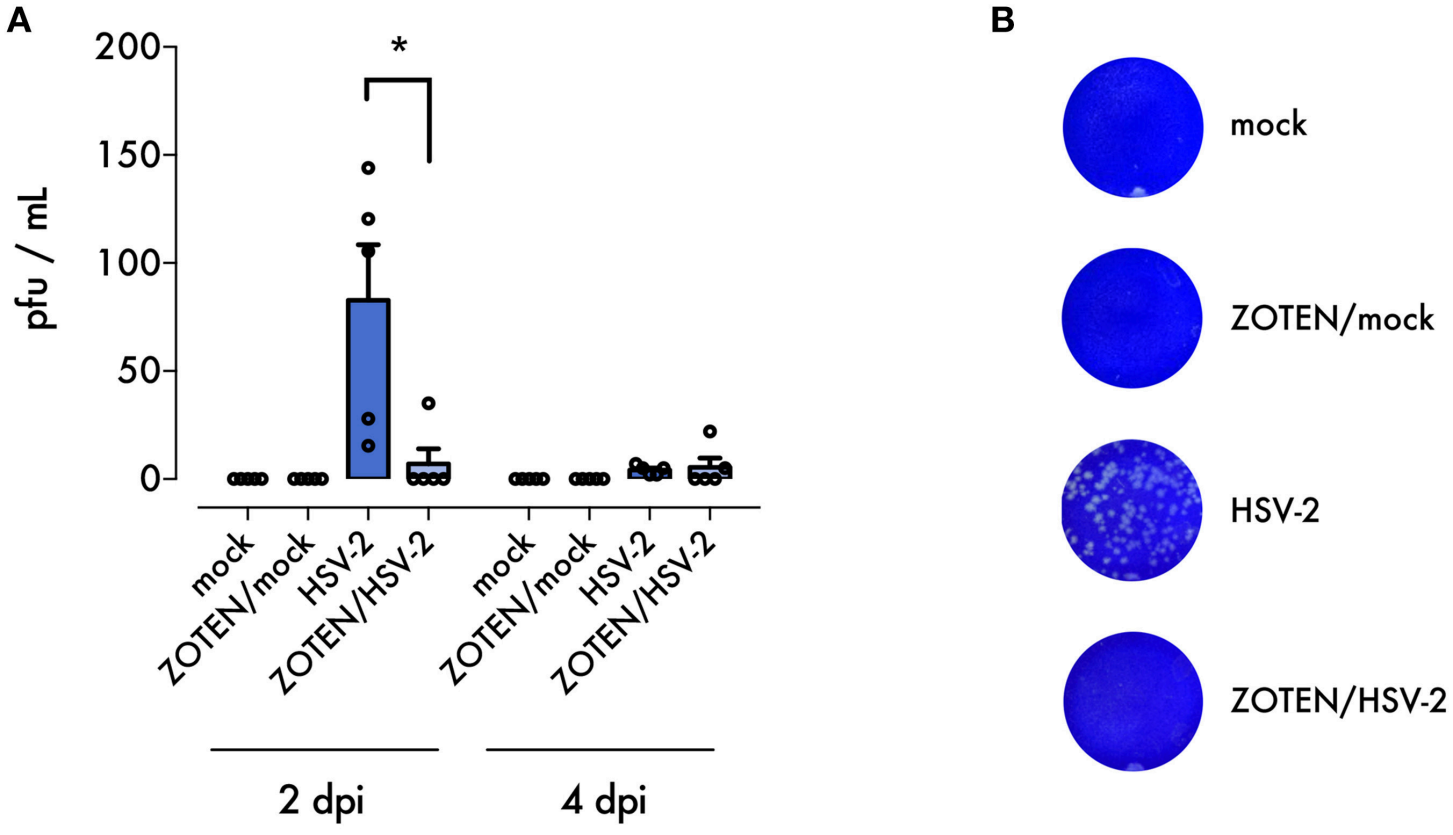
ZOTEN treatment reduces viral shedding. **(A)** Plaque assay results from vaginal swabs at 2 and 4 dpi. Error bars indicate SEM (*n* = 5 per group). Asterisk denotes significant difference by two-tailed unpaired Student's *t*-test, *p < 0.05, ns, or unlabeled, not significant. **(B)** Representative images of crystal violet stained plaque assay results. Zones of clearing are noted in samples from untreated HSV-2 infected mice, indicating presence of replicating virus.

In order to maximize the virus neutralization potential of ZOTEN and study its antiviral and immune benefits we decided to generate a ZOTEN/HSV-2 cocktail by incubating the virus [5 × 10^5^ PFU of HSV-2 (strain 333)] with ZOTEN for 30 min. ZOTEN/HSV-2 was then used for the intravaginal infection of BALB/c mice. To study the effects of the cocktail we created 4 treatment groups of mice: HSV-2 infected, mock infected, ZOTEN/HSV-2 infected and ZOTEN/mock infected (Figure 1). The animals were monitored daily and the antiviral effects were measured for the next 7 days. To determine the presence of productive virus at the primary site of infection and local shedding of infectious virions, vaginal swabs were collected following genital infection with the 4 groups mentioned above. As shown in Figure 2, the viral titers recovered from these vaginal swabs were significantly lower in ZOTEN/HSV-2 group at 2 days post infection, with 4 out of 5 mice displaying no detectable virus. These findings confirm the potent antiviral activity displayed by ZOTEN and its ability to neutralize virus and decrease viral shedding as early as 2 days post infection (Figures 2A,B).

### ZOTEN/HSV-2 Infection Restricts Local Inflammation and Cell Infiltration in Vaginal Tissue

**Figure 3:**
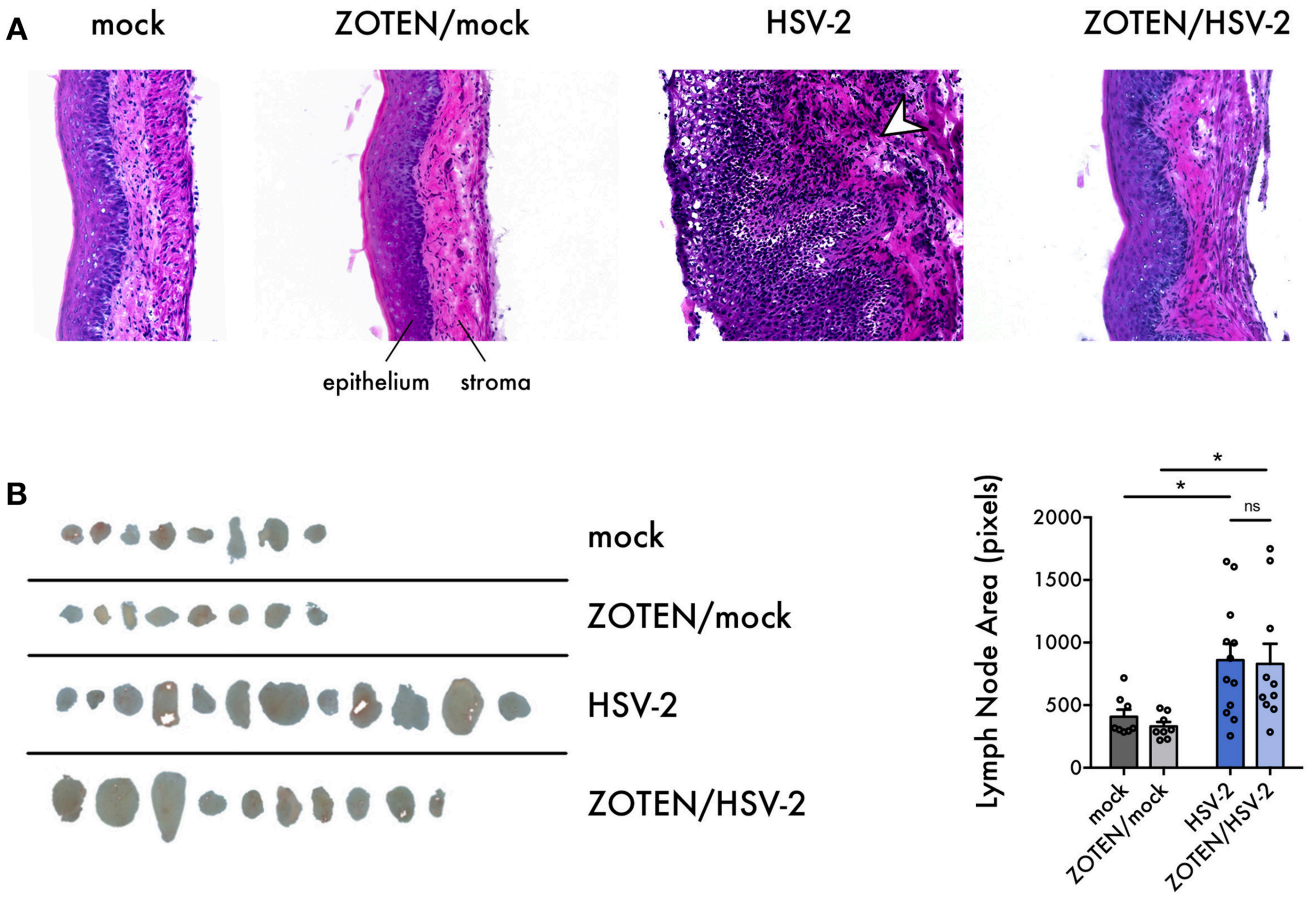
Histological characterization of HSV-2 infection and ZOTEN treatment. **(A)** Representative images of hematoxylin and eosin stained vaginal tissue sections harvested from mice at 7 days post infection. Tissue epithelium and stroma are indicated. Arrowhead in HSV-2 panel indicates tissue infiltration and inflammation observed in infection. Images taken at 20X magnification. **(B)** Draining lymph nodes extracted at 7 days post infection. Areas of lymph nodes in pixels are quantified for each group at right. Error bars indicate SEM. Asterisks denote significant difference by two-tailed unpaired Student's *t*-test, *p < 0.05, ns, or unlabeled, not significant.

To assess disease development, tissue inflammation or damage at the primary site of infection, vaginal tissue was excised at 7 days post infection and analyzed by three methods: histology, quantitative polymerase chain reaction (qPCR) and flow cytometry (Figure 1). Hematoxylin and Eosin (H&E) staining of the vaginal tissue was performed to quantify the phenotypic development of infection as well as activation of innate immune response (Figure 3A). It is evident that ZOTEN treated mice exhibit decreased signs of immune cell infiltration and inflammation, developing low or no apparent levels of acute HSV-2 infection. The thickness of the epithelium in ZOTEN/HSV-2 treated vaginal tissue is comparable to mock infected, as opposed to the apparently inflamed epithelium and increased cell infiltration in HSV-2 infected tissue. Looking beyond the primary site of infection, draining lymph nodes were also isolated to give an indication of the extent of the systemic immune response generated in each group. Lymph nodes isolated from HSV-2 infected mice were apparently larger than those of mock infected mice, regardless of whether they received ZOTEN treatment (Figure 3B).

### ZOTEN/HSV-2 Infected Female Mouse Genitalia Show Signs of Reduced Local Immune Response

**Figure 4:**
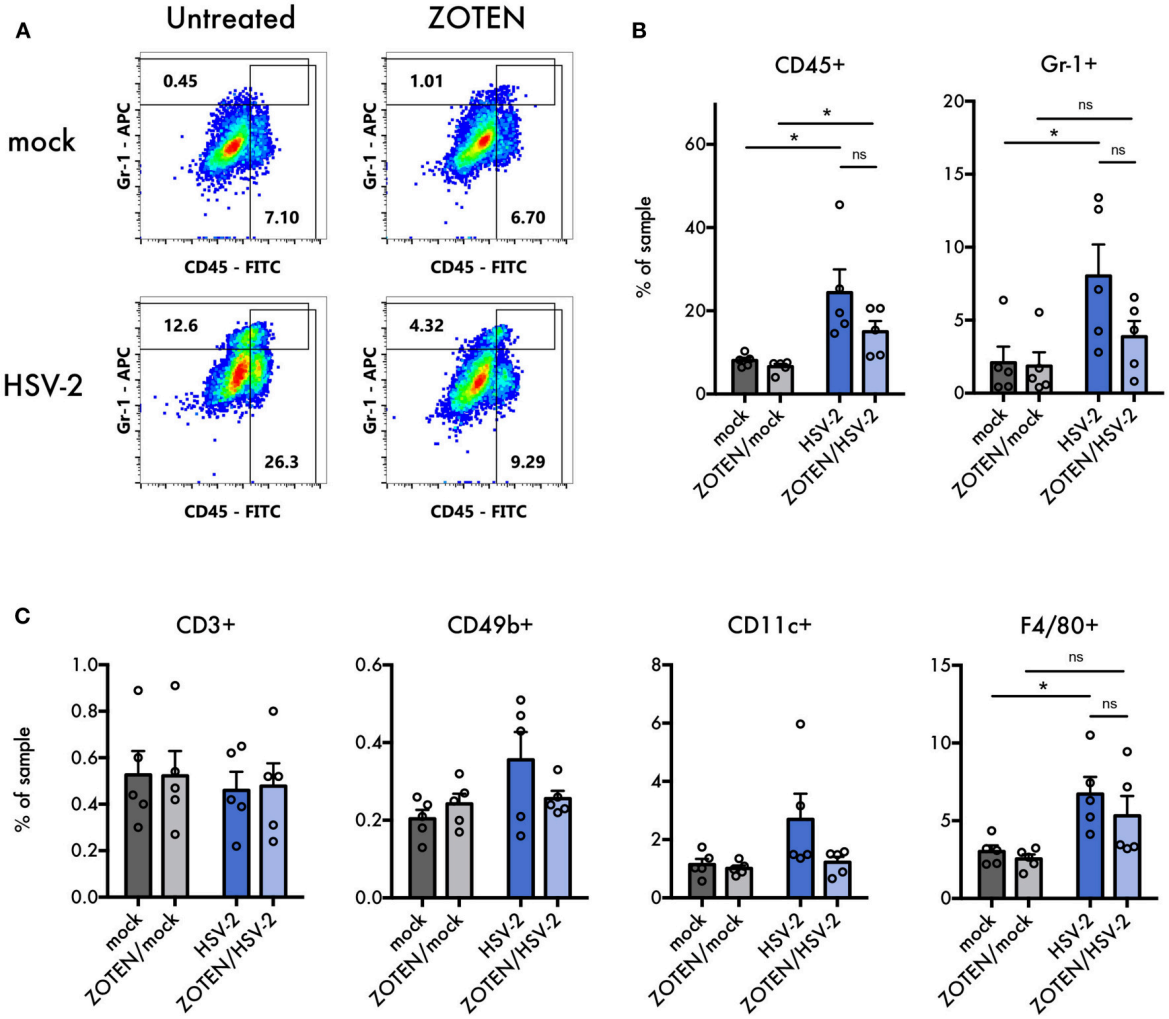
Flow cytometry analysis of immune cell infiltration into vaginal tissues. **(A)** Representative flow cytometry plots of APC anti-Gr-1 vs. FITC anti-CD45. Values in gated regions indicate percentages of singlet non-debris events. **(B)** Quantification of flow cytometry analysis for CD45^+^ cells and Gr-1^+^ cells in vaginal tissues at 7 dpi. Error bars indicate SEM (*n* = 5 per group). **(C)** Quantification of flow cytometry analysis for CD3^+^, CD49b^+^, CD11c^+^, and F4/80^+^ cells in vaginal tissues at 7 dpi. Error bars indicate SEM (*n* = 5 per group). Asterisks denote significant difference by two-tailed unpaired Student's *t*-test, *p < 0.05, ns, or unlabeled, not significant.

**Figure 5:**
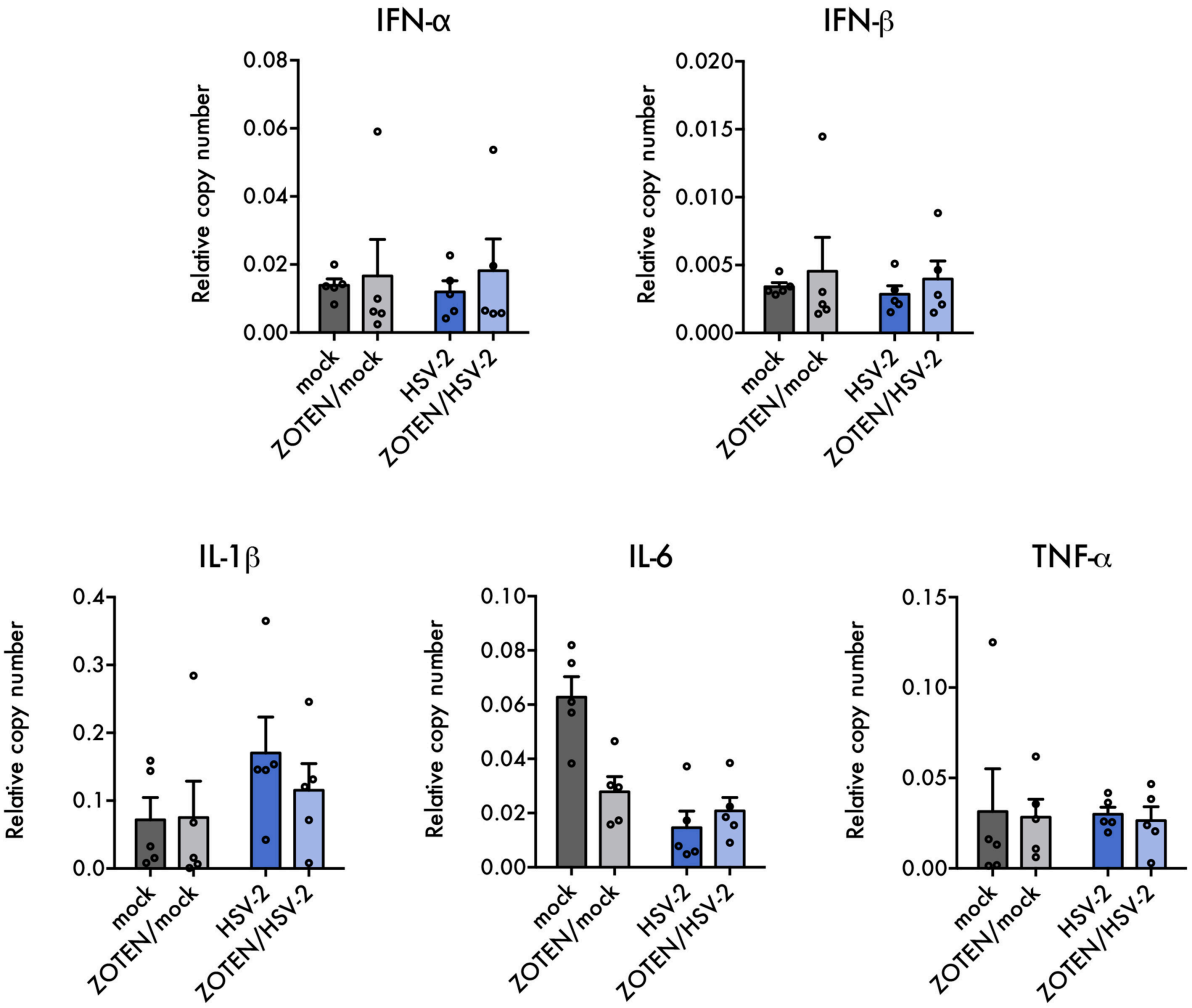
Local cytokine expression in infection and ZOTEN treatment. Quantification of key antiviral type I interferon and pro-inflammatory cytokine transcripts in mice vaginal tissues. Copy numbers relative to b-actin are shown. Error bars indicate SEM (*n* = 5 per group).

Previously published work by our lab demonstrated ZOTEN's ability to exert adjuvant properties by showing increased levels of CD4 and CD8^+^ T cells in isolated splenocytes in response to ZOTEN treatment of HSV-2 infection (24). In this study, we sought to understand the nature of the elicited immune response at the primary site of infection and further identify acute disease development. The isolated vaginal tissues of varying treatment groups were subjected to flow cytometry and the presence of various immune cells were detected (Figure 4). The tissue was stained for CD45, Gr-1, CD3, CD49b, CD11c, and F4/80 positive cells. CD45^+^, Gr-1^+^, and F4/80^+^ cells showed trends of heightened levels in the presence of infection and interestingly displayed a similar trend of decreased levels with ZOTEN/HSV-2 treatment. CD45^+^ cells were significantly higher in HSV-2 infected mice, in comparison to mock infected, as well as ZOTEN/HSV-2 infected mice, in comparison to ZOTEN/mock treatment group. Similarly, Gr-1^+^ cells were detected at significantly higher levels in HSV-2 infected mice when compared to mock infected. A decrease in infiltration of Gr-1^+^ cells was observed between HSV-2 infected and ZOTEN/HSV-2 infected mice and the amount of Gr-1^+^ cells in the vaginal tissue of ZOTEN/HSV-2 infected cells were comparable to mock and ZOTEN/mock infected mice. CD49b^+^ and CD11c^+^ cells increased upon HSV-2 infection but remained at basal levels in the ZOTEN/HSV-2 group. Finally, relatively similar levels of CD3^+^ cells were observed in the four treatment groups. qPCR was also performed on the vaginal tissue to assess levels of pro-inflammatory cytokine transcripts at the local site of infection (Figure 5). While no discernible trends were observed among IFN-α, IFN-β, TNF-α, and IL-6, there was a slight decrease in IL-1β transcript levels in ZOTEN/HSV-2 infected vaginal tissues further supporting the observation of decreased local inflammation (Figure 5).

## Discussion

HSV-2 infection causes significant disease worldwide, putting over 400 million people at risk of increased genital herpes and lifelong viral persistence in latently infected cells (11). HSV-2 most commonly results in painful ulcerations of genital mucosa and skin as well as increased psychological distress among carriers (19). HSV is also capable of infecting the central nervous system resulting in more severe disease development such as meningitis and encephalitis, which in some cases may be fatal (31). More recently, HSV-2 has received more attention as it has been associated with increased risk of HIV acquisition, making it a more relevant and critical virus to study (10, 32, 33). Current HSV-2 treatment options are not optimal as they exhibit problematic features such as developed drug resistance, toxicities and recurrences of infection. The majority of FDA approved drugs target the virus itself and are efficacious in restricting productive viral replication, however they lack the ability to entirely eliminate quiescent viral genomes and therefore cannot prevent reactivation from latency. Evidently, there is a critical need for a protective vaccine or an immunotherapeutic with a novel antiviral mechanism.

Viral survival in the host relies on the ability of the virus to evade host detection of viral determinants, block immediate host antiviral responses and induce responses favorable for its replication and shedding (16, 17). HSV is known to subvert various pathways in the cell such as DNA repair process, type I interferon (IFN) signaling, cell death and proliferation (17). Highly dynamic interactions between replicating HSV-2 and host mediated processes, like local immune responses in genital tissue, contribute to observed disease manifestations and viral persistence. An example observed is the host enzyme, heparanase, which has been identified as a key host protein that drives tissue destruction and viral pathogenesis (34–37). Exploiting tactics used by the virus in the host can provide an effective anti-HSV microbicide.

A microbivac like ZOTEN demonstrates unique and diverse antiviral mechanism that make it a great candidate for further development into a treatment/vaccination for HSV-2 genital infection. We have previously shown that ZOTEN traps the virus, inhibiting viral entry into the cell and simultaneously allowing for detection by immune cells such as antigen presenting cells. ZOTEN enhances anti-HSV-2 immunity and T cell responses and facilitates the development of memory T cells as well as neutralizing antibody response, acting as an immune booster (24, 38).

In this proof-of-concept study, we sought to elucidate short-term tissue specific antiviral efficacy and immune effects of a ZOTEN/HSV-2 cocktail. The cocktail helps to address two important questions. It sheds light on the virus neutralization potential of ZOTEN and more innovatively, shows its promise as a live virus vaccine platform, which reduces infection without compromising local immune responses. We studied the phenotype of infection, pathogenesis and resulting local immune response following genital infection of female BALB/c mice. The elicited immune response by ZOTEN/HSV-2 acts to decrease local cell infiltration and inflammation and therefore results in a global decrease of pathogenesis. To confirm this, we created 4 treatment groups of mice: HSV-2 infected, mock infected, ZOTEN/HSV-2 infected and ZOTEN/mock infected. To maximize our understanding of the events occurring at the primary site of infection, the excised vaginal tissue at 7 days post infection was divided in to 3 equal pieces and each section was subjected to different analysis.

First, we looked at the phenotype of infection by H&E staining (Figure 3A). Representative images of each animal group are shown with evident increased levels of cell infiltration, tissue inflammation and damage in HSV-2 infected mice as opposed to the other treatment groups. The vaginal epithelium of ZOTEN/HSV-2 infected mice was comparable in thickness and morphology as mock infected mice. Interestingly, ZOTEN/HSV-2 infected mice exhibited significantly larger draining lymph nodes than mock infected, comparable to HSV-2 infected mice without treatment, leading us to believe that presence of ZOTEN mediates an immune response similar to non-treated infection. However, ZOTEN treatment more so triggers the development of adaptive immunity and memory against the pathogen (Figure 3B). While further studies are needed, it appears that ZOTEN equips the host with heightened immune surveillance against the virus, allowing it to fight off the infection while minimizing the inevitable side effect of disease development by innate immunity.

In hopes of better understanding the key players contributing to the changes in local immune response upon ZOTEN treatment, we looked at the different types of cells infiltrating the vaginal tissue by flow cytometry. CD45, Gr-1, CD3, CD49b, CD11c, and F4/80 were used as markers for leukocytes, neutrophils, T lymphocytes, natural killer cells, dendritic cells, and macrophages, respectively. A trend of decreased infiltration of CD45^+^, Gr-1^+^, and F4/80^+^ cells was observed in the vaginal tissue upon ZOTEN/HSV-2 genital infection. ZOTEN also restored basal levels of CD49b^+^ and CD11c^+^ cells. It is understood that neutrophils (expressing Gr-1) are a major component of the innate inflammatory infiltrate at the primary site of herpes infection (18). The observed trends of decreased CD45^+^ and Gr-1^+^ infiltrating cells in the vaginal epithelium in addition to lower levels of proinflammatory IL-1β transcripts further demonstrates the decreased local inflammation observed in ZOTEN/HSV-2 infected mice (Figures 4A,B, 5).

Tissue specific analysis of the application of ZOTEN has allowed for better understanding of how the infection is processed in the local tissue environment. HSV-2 infected mice, in the presence or absence of a prior ZOTEN treatment, demonstrate similar levels of activation of the immune system however differ drastically in phenotype of local infection. This leads us to believe that while the immune response is activated in the presence of ZOTEN, local inflammation is limited and therefore clinical manifestations of infection are suppressed. Therefore, ZOTEN acts to bolster the immune system and equip the host with a better response to infection. ZOTEN shows to be a practical solution for instant benefit as a microbicide and future development of vaccine against HSV. In addition, our studies show the promise that ZOTEN can be developed as a live virus vaccine platform whereby the viral candidates for the vaccine can be preincubated with ZOTEN and then delivered via intravaginal or other routes. An optimized combination will not cause infection but elicit a protective and/or therapeutic immune response. While more studies are definitely needed, ZOTEN as a live virus vaccine platform is another out-of-the-box strategy, which may lead to new and more effective vaccine strategies.

## Data Availability

All datasets generated for this study are included in the manuscript and/or the supplementary files.

## Ethics Statement

All animal experiments were reviewed by the UIC Animal Care Committee and the experiments were performed in adherence to the ARVO Statement for the Use of Animals in Ophthalmic and Vision research.

## Author Contributions

AA, LK, RS, TY, and YM performed the experiments. AA, LK, RS, and TY analyzed the results from the biological experiments and YM and RA analyzed the ZOTEN synthesis data. AA, LK, and DS conceived the study and wrote the manuscript.

### Conflict of Interest Statement

The authors declare that the research was conducted in the absence of any commercial or financial relationships that could be construed as a potential conflict of interest.
